# Isolation of Human Platelets (Thrombocytes)

**DOI:** 10.1100/tsw.2002.820

**Published:** 2002-06-12

**Authors:** John Graham

**Affiliations:** School of Biomolecular Sciences, Liverpool John Moores University, UK

**Keywords:** platelets, autologous plasma, thrombocytes, OptiPrep™, iodixanol, density barrier, sedimentation velocity

## Abstract

Platelets from human blood can be isolated in high yield by centrifugation of whole blood over an iodixanol density barrier of 1.063 g/ml. The separation from all of the blood cells (which form a pellet) is based on the slower sedimentation velocity of the smaller platelets.

**METHOD TYPE:** extraction, isolation, purification and separation

**SUB METHOD TYPE:** centrifugation

## INTRODUCTION

Although platelet-rich plasma (PRP) is relatively easy to produce by centrifugation of whole blood, yields of platelets may be variable, because many of them are trapped within the erythrocyte layer. Although they can be recovered by washing these cells with isotonic saline, it is a general rule that to avoid activation of the platelets, the number of centrifugations and resuspensions should be kept to a minimum. Another problem is that aspiration of the PRP must be performed carefully to avoid contamination from leucocytes in the buffy coat, which lies atop the erythrocytes.

To provide a highly purified platelet fraction from human blood Ford et al.[1] layered whole blood over a density barrier of Nycodenz ® ($\rho$ = 1.063 g/ml) that allowed the erythrocytes and leucocytes to pellet during centrifugation at 350*g*. The platelets, because of their small size, sediment much more slowly; they were recovered as a broad band extending into the density barrier from just above the interface.

The platelets recovered from this density barrier method were used directly in aggregation studies, the Nycodenz® did not interfere with this process[1].

An iodixanol barrier of the same density can be substituted for the Nycodenz®; the separation of platelets, their yield, and functional competence are the same.

## MATERIAL AND EQUIPMENT

OptiPrep™
Diluent: 0.85% (w/v) NaCl, 20 mM Hepes-NaOH, pH 7.4, 1 mM EDTA

Plastic conical centrifuge tubes (12–15 ml)
Plastic Pasteur pipette for overlayering
Low-speed (temperature-controlled) centrifuge with swinging-bucket rotor

## METHOD

1. Collect blood by venepuncture into a suitable anticoagulant (EDTA or citrate).
2. Gently shake the contents of the OptiPrep™ bottle before use.
3. Produce the $\rho$ = 1.063 g/ml density barrier by mixing 5 vol OptiPrep™ with 22 vol of the Diluent (see Note 1).
4. In a centrifuge tube layer 1 vol of blood over 1 vol of density barrier (see Notes 2 and 3) and centrifuge at 350*g* for 15 min at 20°C in a swinging-bucket rotor and allow the rotor to **decelerate without the brake.**
5. Harvest the autologous plasma and the platelet-containing band as shown in Fig. 1.
6. Towards the bottom (lowest 2–3 mm) of the platelet band there will be a slight increase in the contamination by leucocytes and erythrocytes (up to 3–5%) while the contamination in the bulk of the platelet band is <1%.

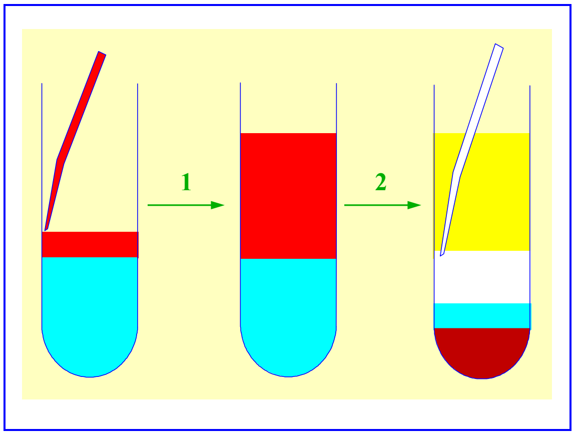

**FIGURE 1**. Isolation of platelets from whole human blood. (1) Equal volumes of blood and density barrier layered in tube. (2) After centrifugation platelets harvested from the broad turbid band below the interface.

## NOTES

1. See Ref. 2 for more information on preparing density solutions.
2. The separation of the platelets is based on their slow rate of sedimentation, so it is very important that the centrifugation speed and time is carefully adhered to. Higher speeds and longer times will result in the platelet band moving closer to the cell pellet.
3. To permit an adequate linear separation of the platelets from the pellet the density barrier column needs to be approx. 5 cm, thus in a 15-ml centrifuge tube there should a minimum of 5 ml of density barrier. For small volumes (1–5 ml of blood) use 5 ml of barrier; for larger volumes of blood, use an equal volume of barrier. The column height of the density barrier in larger volume tubes should be maintained.

## ACKNOWLEDGEMENTS

The author and TheScientificWorld wish to thank Axis-Shield PoC, AS, Oslo, Norway for their kind permission to adapt OptiPrep™ Application Sheets C12 in the preparation of this Protocol Article.
